# Pandemic fatigue and associated factors: a meta-analysis using the COM-B model

**DOI:** 10.3389/fpsyg.2026.1765375

**Published:** 2026-04-23

**Authors:** Jin Zhao, Jiayu Wang, Luo Jiang, Fuxian Li, Lili Ji, Hui Jin

**Affiliations:** 1 Key Laboratory of Environmental Medicine Engineering, Ministry of Education, Department of Epidemiology & Biostatistics, School of Public Health, Southeast University, Nanjing, China; 2 Nanjing University of Chinese Medicine, Nanjing, China; 3 Jiangsu Provincial Center for Disease Control and Prevention, Nanjing, China

**Keywords:** COM-B model, COVID-19, meta-analysis, pandemic fatigue, TDF

## Abstract

**Background:**

Pandemic fatigue during the prolonged COVID-19 crisis undermines sustained engagement with protective measures and public health messaging. Existing studies provide heterogeneous prevalence estimates and disparate lists of correlates but lack a unified, theory-driven synthesis.

**Methods:**

Searches were conducted in six databases up until March 2026. This study synthesized the prevalence of pandemic fatigue and factors associated with it during COVID-19 using the COM-B model and TDF theory.

**Results:**

Twenty-three cross-sectional studies (*n* = 49,285) were included. The pooled prevalence of pandemic fatigue was 51% (95%CI: 0.38, 0.65), with substantial heterogeneity. Health literacy (Capability) was inversely associated with fatigue (*β* = −0.257, 95%CI: −0.404, −0.110). Opportunity-related stressors—including bereavement due to COVID-19 (*β* = 0.281, 95%CI: 0.124, 0.437), daily troubles (*β* = 0.296, 95%CI:0.211, 0.380), and working student status (*β* = 0.232, 95%CI: 0.122, 0.341)—were positively associated with pandemic fatigue. Motivation-related factors showed mixed associations, whereas negative emotional states were associated with higher odds of pandemic fatigue.

**Conclusion:**

Pandemic fatigue is common and was associated with diverse capability-, opportunity-, and motivation-related factors. A COM-B-informed interpretation suggests multi-level strategies that combine skills building, supportive environments, and psychosocial support.

## Introduction

Since the coronavirus disease (COVID-19) outbreak in 2019, social structures and lifestyles worldwide have undergone unprecedented changes. People have to contend not only with ongoing health threats but also with the widespread effects of prolonged lockdowns, social isolation, and economic stress (Emodi-Perlman et al., 2021; Huang et al., 2021). In this context, the phenomenon of “pandemic fatigue” has gradually attracted significant attention. Pandemic fatigue not only reflects the psychological exhaustion resulting from a prolonged health crisis but also highlights the challenges societies face in their enduring response strategies (Meacci and Primicerio, 2021).

Pandemic fatigue has been described as a natural response to persistent and unresolved adverse events. It manifests as emotional distress, diminished motivation to engage in protective behaviors, reduced information seeking, and feelings of complacency, alienation, and hopelessness. Fatigue evolves and is influenced by various emotions and experiences, perceptions and the broader cultural, social, structural, and legislative environments (World Health Organization, 2020). Pandemic fatigue, particularly as it relates to compliance with COVID-19 restrictions, is a critical issue affecting pandemic response, necessitating a definition that encompasses behavioral fatigue (Gountas et al., 2021).

Pandemic fatigue has been operationalized in related literature through overlapping manifestations, particularly information fatigue and behavioral fatigue (World Health Organization, 2020; So et al., 2017; Su et al., 2022). Information fatigue or overload occurs when individuals feel overwhelmed by excessive information, leading to confusion and decision-making difficulties (Cao et al., 2021). During the pandemic, frequent information updates and extensive media coverage made the public susceptible to information fatigue (Yin et al., 2024), which can reduce their motivation to maintain protective behaviors (Wu et al., 2024) and may further jeopardize the effectiveness of public health messages (Steiner et al., 2021).

Therefore, in this study, pandemic fatigue was operationally defined as a broad fatigue response under prolonged pandemic conditions. Under this definition, information fatigue and behavioral fatigue were treated as related manifestations of a broader construct rather than mutually exclusive categories. It was reflected in prolonged exposure to pandemic-related information, public health measures, or pandemic-related restrictions, and may manifest as emotional exhaustion, reduced motivation for protective behaviors, or information overload. Such manifestations have been discussed in relation to factors such as information overload (Sun and Lee, 2023), social isolation (Campos et al., 2022), and public health policies (Yang et al., 2024).

To effectively distinguish and classify heterogeneous factors associated with pandemic fatigue, it is essential to use the appropriate health behavior theory (Michie et al., 2005). The Capability, Opportunity, and Motivation–Behavior (COM-B) model is a widely used theoretical framework for understanding the factors that influence behavior (Michie et al., 2014). As a core component of the Behavior Change Wheel, which incorporates 19 behavioral theories, the COM-B model posits that three holistic factors are necessary for any behavior to occur: motivation (automatic and reflective), capability (physical and psychological), and opportunity (social and physical). Each factor’s subcomponents can act as barriers or drivers of behavior (Michie et al., 2011), contributing to a comprehensive understanding of behavioral change and aiding in the design of effective interventions.

Given that pandemic fatigue involves both psychological depletion and behavioral disengagement, the COM-B model offers a comprehensive framework to understand factors associated with it. Specifically, pandemic fatigue may reflect reduced capability to process health information, constrained opportunity under prolonged social and structural stress, and diminished motivation to sustain protective responses. This mapping allows a finer-grained analysis of how specific psychosocial factors relate to pandemic fatigue. The Theoretical Domains Framework (TDF) further refines the COM-B components by providing more specific theoretical domains, thereby supporting a more systematic and transparent classification of heterogeneous psychosocial variables reported across studies.

A detailed understanding of the current status of pandemic fatigue and the factors associated with it is critical for developing comprehensive and effective interventions to mitigate pandemic fatigue (Stamm et al., 2023). This study aimed to systematically integrate and evaluate existing studies through meta-analysis, identify factors associated with pandemic fatigue, and map them to the components of the COM-B model. Given the centrality of the COM-B model within the BCW, it can guide researchers and policymakers in developing targeted strategies and provide a scientific basis for more effective public health policies and interventions (Mersha et al., 2020). Our research offers valuable insights for optimizing future outbreak response strategies, providing better protection, and promoting the mental and social health of the global population during current and future health crises.

## Method

This meta-analysis was registered with PROSPERO (CRD42024576613) and followed the Preferred Reporting Items for Systematic Reviews and Meta-Analyses (PRISMA) guidelines (Page et al., 2021).

### Search strategy

A comprehensive search was conducted across multiple databases, including Chinese and English sources such as the China National Knowledge Infrastructure (CNKI), Wanfang Database, PubMed, Embase, Web of Science, and Scopus. We searched records published between January 1, 2020, and March 11, 2026. The search started in January 2020 to ensure sensitivity and capture early pandemic-related fatigue research using varied terminology. However, formal eligibility was restricted to studies published after October 2020, when WHO explicitly introduced the term “pandemic fatigue.” Search terms included a combination of subject terms and free-text terms related to pandemic fatigue and conceptually overlapping fatigue manifestations, including pandemic fatigue, vaccine fatigue, information fatigue, behavioral fatigue, immune fatigue, and message fatigue. Detailed search strategies for all databases are provided in Supplementary material S2. COVID-19 relevance was further determined during title/abstract screening and full-text eligibility assessment.

In the search strategy, two core concepts were identified *a priori*: (1) pandemic fatigue and related fatigue manifestations, and (2) the pandemic context. Keywords and alternative expressions were generated from the WHO terminology on pandemic fatigue, related literature, and terms identified during preliminary scoping of relevant studies. Synonyms within the fatigue-related concept (e.g., “pandemic fatigue,” “vaccine fatigue,” “information fatigue,” “behavioral fatigue,” “immune fatigue,” and “message fatigue”) were combined using the Boolean operator OR. Phrase searching with quotation marks was used, and database-specific field tags were adapted according to each platform. Because the term “pandemic fatigue” itself directly reflects the pandemic context, and because some related fatigue expressions were used variably across studies, the search strategy prioritized sensitivity by using broad fatigue-related terms at the database level, while COVID-19 relevance was further confirmed during title/abstract screening and full-text eligibility assessment.

### Inclusion and exclusion criteria and outcome definitions

To select studies, we applied predetermined Population, Intervention, Comparator, Outcome, Time, and Study design (PICOTS) criteria. In the implementation of PICOTS, the Outcome component was reflected most directly in the search strategy through fatigue-related terms, including pandemic fatigue and conceptually related manifestations. The pandemic context was partially captured by the term “pandemic fatigue” itself and was further confirmed during screening and full-text eligibility assessment. Population (adults), study design (quantitative observational studies), and publication time restrictions were primarily implemented during the screening stage rather than through separate database search blocks. Comparator was not used as an independent search component because comparison groups were not defined uniformly across the included observational studies. Intervention was not implemented as an independent search component because this review focused on observational studies of pandemic-related fatigue rather than intervention effects.

Inclusion criteria:

1. Participants must be adults (≥18 years of age) who have experienced the COVID-19 pandemic.
2. Eligible studies assessed pandemic fatigue as reflected in information fatigue (related to pandemic-related information), behavioral fatigue (reduced willingness to take protective action), and/or emotional or cognitive exhaustion related to prolonged pandemic conditions. Studies were eligible if they assessed at least one of these manifestations; co-occurrence of all manifestations was not required.
3. Studies must use valid scales or questionnaires that explicitly report pandemic fatigue measurements (total participants, number of participants with fatigue and fatigue prevalence) or explore psychological or behavioral factors associated with pandemic fatigue and provide quantifiable results (mean, standard deviations, or regression coefficients) for meta-analysis.
4. Quantitative studies had to be peer-reviewed and provide sufficient data for extraction and analysis.
5. Only studies published after October 2020, when WHO officially introduced pandemic fatigue, focused on data from COVID-19 and related pandemics.

Exclusion criteria:

1. Studies involving children or minors or targeting specific occupational groups (e.g., medical personnel) unless part of a larger sample.
2. Qualitative studies, critical reviews, conference abstracts, editorials, case reports, and non-peer-reviewed literature.
3. Studies with incomplete data or missing essential statistics, particularly those lacking quantitative measurements of fatigue or relevant associated factors.
4. Duplicate studies.

Following Michie’s framework, TDF was used to operationalize the COM-B components, with TDF domains mapped to the three constructs—Capability (knowledge, behavioral regulation), Opportunity (social influences, environmental context), and Motivation (beliefs, emotions). This mapping allows a finer-grained analysis of how specific psychosocial factors are associated with pandemic fatigue.

The COM-B model was selected because pandemic fatigue involves both psychological exhaustion and reduced engagement in protective behaviors, making it necessary to adopt a framework that captures multiple influences on behavior. The TDF further complements the COM-B model by providing a set of theoretical domains that enable a more detailed examination of psychosocial influences, including knowledge, social influences, emotions, and environmental context. Using these frameworks together allows for a systematic identification of barriers and facilitators related to pandemic fatigue and provides a theoretical basis for interpreting the observed associations. This framework also supported the inclusion of studies using different but conceptually overlapping operationalizations of pandemic fatigue. In regression-based studies, pandemic fatigue was treated as the outcome variable, whereas capabilities, opportunities, and motivations related to it were treated as explanatory variables.

Accordingly, the primary explanatory variables were those derived from the COM-B and TDF domain coding manuals. The findings related to the facilitating factors and barriers outlined in the TDF and those identified in the study were coded independently by two authors, with the final code reviewed by a senior author (Michie et al., 2005; Cane et al., 2012). Odds ratios (ORs) and beta coefficients (*β*) were synthesized separately and were not pooled together. For regression-based studies reporting *β* coefficients, unstandardized *β* coefficients were extracted whenever available. When both standardized and unstandardized coefficients were reported, the unstandardized coefficients were selected for consistency. To maintain comparability across studies, unadjusted estimates were extracted for pooling. Only coefficients referring to conceptually comparable variables with consistent coding direction were pooled. Where comparability was limited by differences in variable operationalization or measurement scales, the pooled estimates were interpreted cautiously.

### Data extraction

Two reviewers independently screened the literature and crosschecked the extracted data. Discrepancies were resolved through consultation with a third party, and studies with incomplete data were excluded. The extracted data included title, first author, year of publication, study location, sample size, pandemic fatigue assessment tools, and factors associated with pandemic fatigue.

### Quality appraisal

Study quality was assessed after full-text inclusion and was used to describe methodological quality and support interpretation of the findings, rather than as an *a priori* screening criterion. For cross-sectional studies, we used data from the U.S. Agency for Healthcare Quality and Research (AHQR) (Stang, 2010). The AHQR tool evaluates aspects of study design, sample selection, data collection, and result analysis to assess the internal validity and external generalizability of cross-sectional studies. For cohort studies, we used the Newcastle-Ottawa Scale (NOS). This scale assesses the potential risk of bias by evaluating the quality of the selection, comparability, and outcome assessment. Specifically, it focuses on sample representation, identification of exposure factors, measurement of outcomes, and the integrity of follow-up. No studies were excluded solely on the basis of quality appraisal scores.

### Data analysis

Data extraction and meta-analysis were performed using Microsoft Excel 2019 and STATA 14.0.

In the meta-analysis, the prevalence was pooled, and heterogeneity was assessed using *I*^2^. Because the included studies used conceptually overlapping but psychometrically non-identical measures, the prevalence meta-analysis was intended to approximate the burden of broadly defined pandemic-related fatigue manifestations rather than the exact prevalence of a single psychometrically uniform construct. Forest plots were used to present odds ratios (ORs), beta coefficients (*β*) and 95% confidence intervals (95% CI) for factors associated with pandemic fatigue across the Capability, Opportunity, and Motivation domains. *I*^2^ statistics were used to describe inter-study heterogeneity. If *I*^2^ ≤ 50%, a fixed effect model was used; If *I*^2^ > 50%, a random-effects model was used. A sensitivity analysis was performed by sequentially excluding individual studies to test the robustness of the results. Correlates were categorized according to the domains of the Theoretical Domains Framework (TDF), and pooled OR/*β* values were estimated for individual TDF domains as well as for the broader Capability (C), Opportunity (O), and Motivation (M) categories. Given the uncertainty in the relative weighting of individual variables, all eligible studies were retained when pooling ORs and *β* coefficients within the TDF domains and the broader C, O, and M categories.

## Results

### Characteristics of the included literature

**Figure 1:**
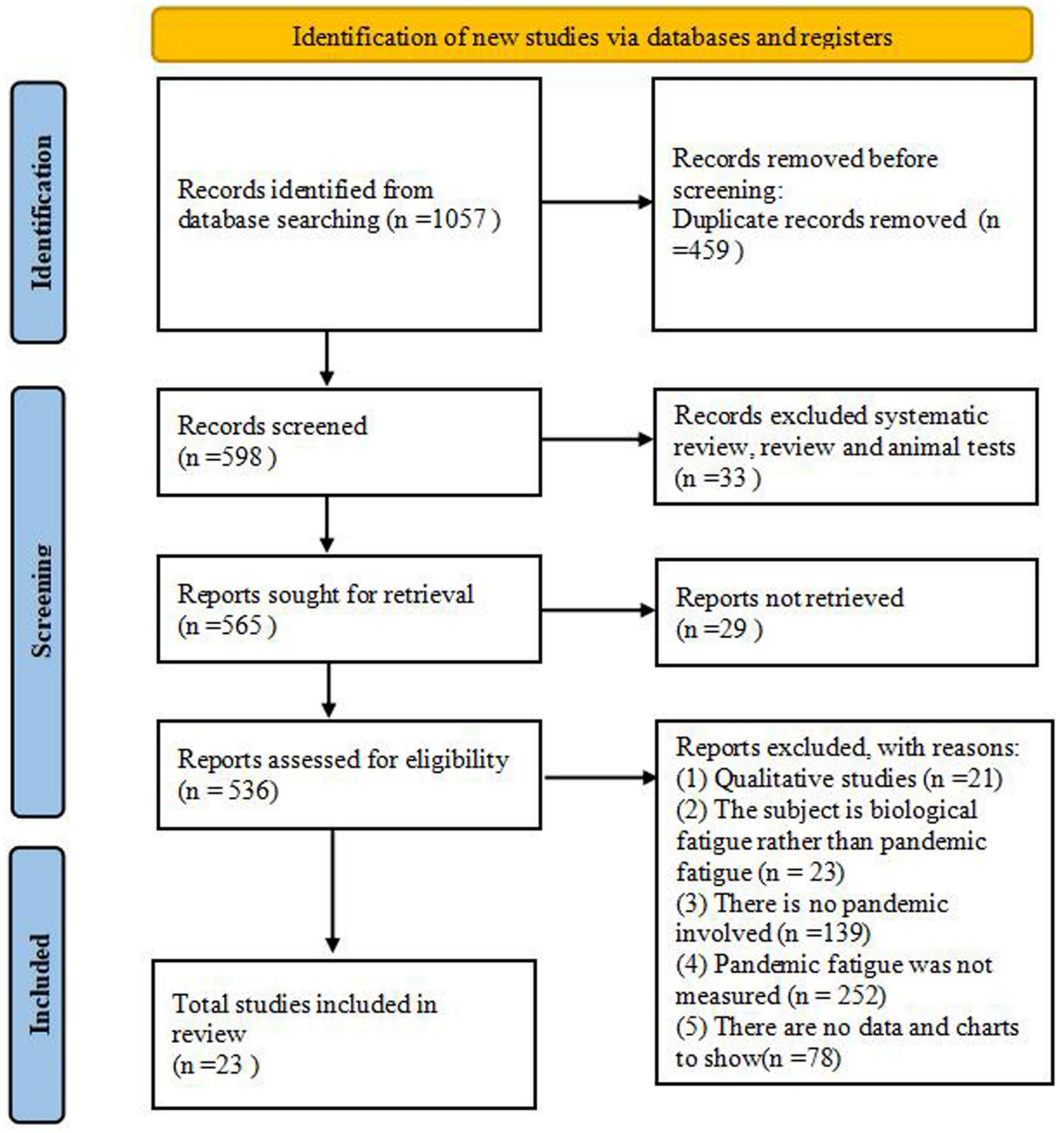
Flow chart of study selection.

The search yielded 1,057 records, of which 23 studies met eligibility criteria and were included (Figure 1). These cross-sectional studies sampled a total of 49,285 adults across multiple countries (China, Malaysia, the Philippines, Spain, Saudi Arabia, India, Tunisia, Turkey, and the United States) between 2020 and 2026, primarily via online surveys and telephone interviews. Study quality scores ranged from 4 to 8 (mean = 5.7); details are provided in Table 1 and Supplementary Table S1.

**Table 1:** Characteristics of the included literature.

Author and published year	Country	Study period	Study type	Sample size	Pandemic fatigue prevalence	Measurement tool	Assessment of factors associated with pandemic fatigue	Way of release	Quality assessment score
Liu et al. (2023)	China	February–March 2021	Cross-sectional study	4,325	25%	Self-prepared questionnaire	Yes	Online	4
Abdul Rashid et al. (2023)	Malaysia	April 1 to April 30, 2022	Cross-sectional study	775	54%	Fatigue Assessment Scale (FAS)	Yes	Online	4
Xin et al. (2022)	China	January–February 2022	Cross-sectional study	1,354	49%	The Pandemic Fatigue Scale (PFS)	Yes	Online	6
Zhang et al. (2024)	China	January–February 2023	Cross-sectional study	479	82%	Fatigue Assessment Scale (FAS)	Yes	Online	7
Leung et al. (2022)	China	February 22 to March 23 2021	Cross-sectional study	4,726	43%	A single direct question	Yes	Online	6
Guan et al. (2023)	America	November 10 to December 10 2020	Cross-sectional study	744	67%	Message Fatigue Scale	No	Online	7
Qin et al. (2023)	China	July 4 to August 11 2023	Cross-sectional study	2,942	73%	The Pandemic Fatigue Scale (PFS)	No	Online	8
Zhao et al. (2024)	America	From August 11 to September 13 2022	Cross-sectional study	16,546	41%	Message Fatigue Scale	Yes	Online	7
Yan et al. (2022)	China	From December 2020 to January 2021	Cross-sectional study	1,255	26%	Fatigue Assessment Scale (FAS)	Yes	Telephone survey	7
Cleofas and Oducado (2021)	The Philippines	June in 2021	Cross-sectional study	1,467	/	Pandemic Fatigue Scale (PFS)	Yes	Online	7
Cleofas and Oducado (2022)	The Philippines	July in 2021	Cross-sectional study	1,665	/	Pandemic Fatigue Scale (PFS)	Yes	Online	4
González-Herrera et al. (2022)	Spain	From January to June 2021	Cross-sectional study	3,005	/	Pandemic Fatigue Scale (PFS)	Yes	Online	5
Hassanien et al. (2022)	Saudi Arabia	April to June 2021	Cross-sectional study	650	/	PF Scale	Yes	Online	5
Kumar et al. (2022)	India	After the end of the second wave of coronavirus in India	Cross-sectional study	256	/	Pandemic/Lockdown Fatigue Scale	Yes	Online	4
Labrague (2021)	The Philippines	January–February 2021	Cross-sectional study	255	/	Pandemic Fatigue Questionnaire	Yes	Online	5
Lai et al. (2023)	China	December 2021 to January 2022	Cross-sectional study	803	/	Fatigue Assessment Scale (FAS)	Yes	Telephone survey	4
Haktanir et al. (2022)	Turkey	November 1 to November 12, 2020	Cross-sectional study	500	/	The COVID-19 Burnout Scale	Yes	Online	6
Cleofas et al. (2021)	The Philippines	The first week of July 2021	Cross-sectional study	1,190	/	Pandemic Fatigue Scale (PFS)	Yes	Online	5
Zhuang et al. (2025)	China	April to May 2022	Cross-sectional study	2024	/	Pandemic Fatigue Scale (PFS)	Yes	Online	6
Kotti et al. (2025)	Tunisia	December 2021 to March 2022	Cross-sectional study	261	/	Pandemic Fatigue Questionnaire	Yes	Online	5
Leiter et al. (2025)	America	April 6 and June 2 of 2022	Cross-sectional study	250	/	Fatigue Assessment Scale (FAS)	Yes	Online	5
Yu et al. (2025)	China	November to December 2024	Cross-sectional study	2,604	/	Self-prepared questionnaire	Yes	Online	6
Wang et al. (2026)	China	January to February 2022	Cross-sectional study	1,209	/	Pandemic Fatigue Scale (PFS)	Yes	Online	7

### Prevalence

**Figure 2:**
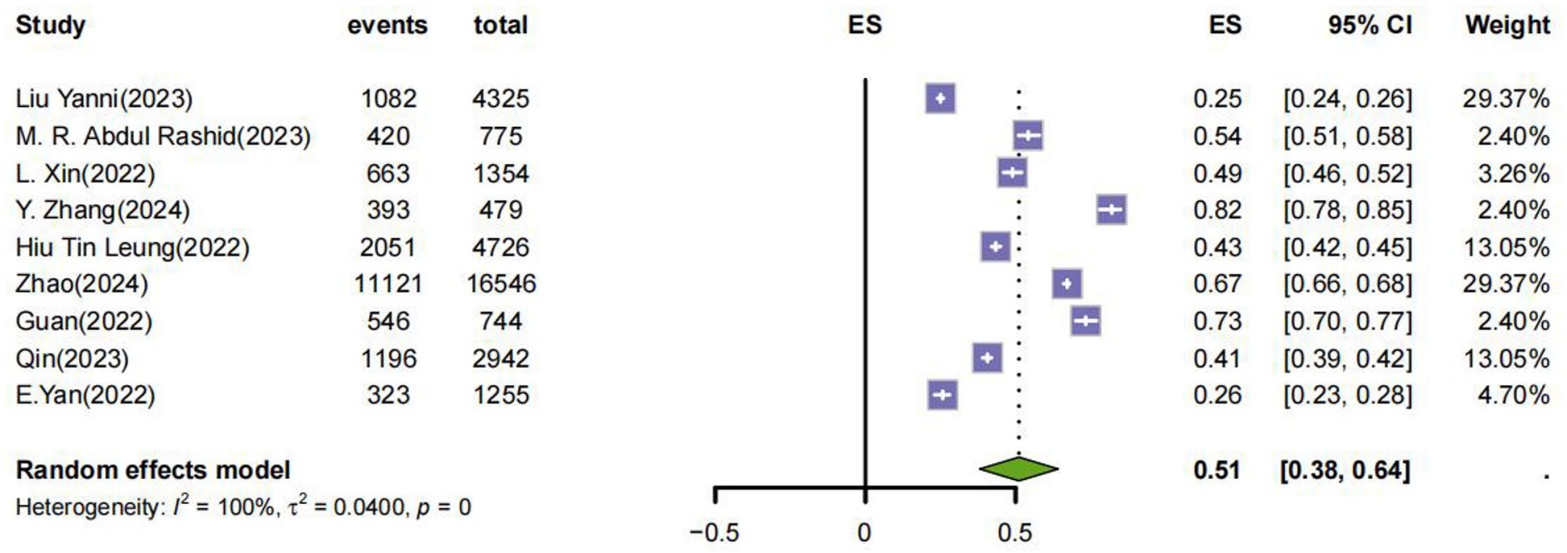
Status of pandemic fatigue.

Nine eligible studies reported prevalence rates, indicating significant heterogeneity (Figure 2). Using a random-effects model, the combined prevalence of pandemic fatigue was 51% (95%CI: 0.38, 0.65). Publication bias was assessed using Egger’s test (*p =* 0.391), which did not indicate significant bias; however, given the limited number of studies, this result should be interpreted cautiously. Given the substantial conceptual and measurement heterogeneity across instruments, this pooled estimate should be interpreted cautiously.

We performed a subgroup analysis, as shown in Table 2. In a country-based subgroup analysis, the United States appeared to have a relatively high prevalence of pandemic fatigue compared with other countries. However, subgroup estimates based on very small numbers of studies should be interpreted as descriptive and exploratory rather than as robust between-group comparisons.

**Table 2:** Subgroup analysis of the pandemic fatigue prevalence.

Subgroup	Number of documents	Total number	Number of cases	Prevalence	95%CI	*I* ^2^	*Z*	*p*
Country								
China	6	15,081	5,708	44.2%	0.320–0.565	99.599%	7.094	<0.01
America	2	17,290	11,667	67.5%	0.668–0.682	/^a^	189.622	<0.01
Malaysia	1	775	420	54.2%	0.506–0.577	/	7.383	<0.01
Questionnaires								
FAS^b^	3	2,509	1,136	54.0%	0.203–0.877	/	3.140	<0.01
MFS^c^	2	19,488	12,317	63.5%	0.628–0.642	/	187.600	<0.01
PFS^d^	2	2,098	1,209	59.0%	0.570–0.611	/	56.722	<0.01
Custom questions	2	9,051	3,133	33.4%	0.324–0.343	/	68.646	<0.01
Time								
2020–2021	4	26,852	14,577	40.4%	0.171–0.636	99.925%	3.402	<0.01
2022–2023	5	6,294	3,218	59.8%	0.443–0.753	99.363%	7.571	<0.01

a In subgroup analysis, the number of subgroup studies after grouping was 3 or less, and the results of heterogeneity test were not displayed.

b Fatigue Assessment Scale.

c Message Fatigue Scale.

d Pandemic Fatigue Scale.

Mapping pandemic fatigue factors onto the COM-B behavioral system.

The mapping analysis based on COM-B suggests a behavioral system underlying pandemic fatigue: inadequate health literacy (capability) reduces individuals’ confidence to manage information, whereas adverse environments and social losses (opportunity) amplify external stressors. These interact with motivational depletion, such as emotional exhaustion and weakened resilience, to jointly drive behavioral withdrawal and cognitive disengagement from pandemic-related information.

### Associated factors

The associated factors of each data-supported lifestyle behavior for pandemic fatigue mentioned in the included studies were extracted, reflected in the TDF domains, and classified into their respective COM-B domains, as shown in Supplementary Table S3.

### COM-B domain classification

**Figure 3:**
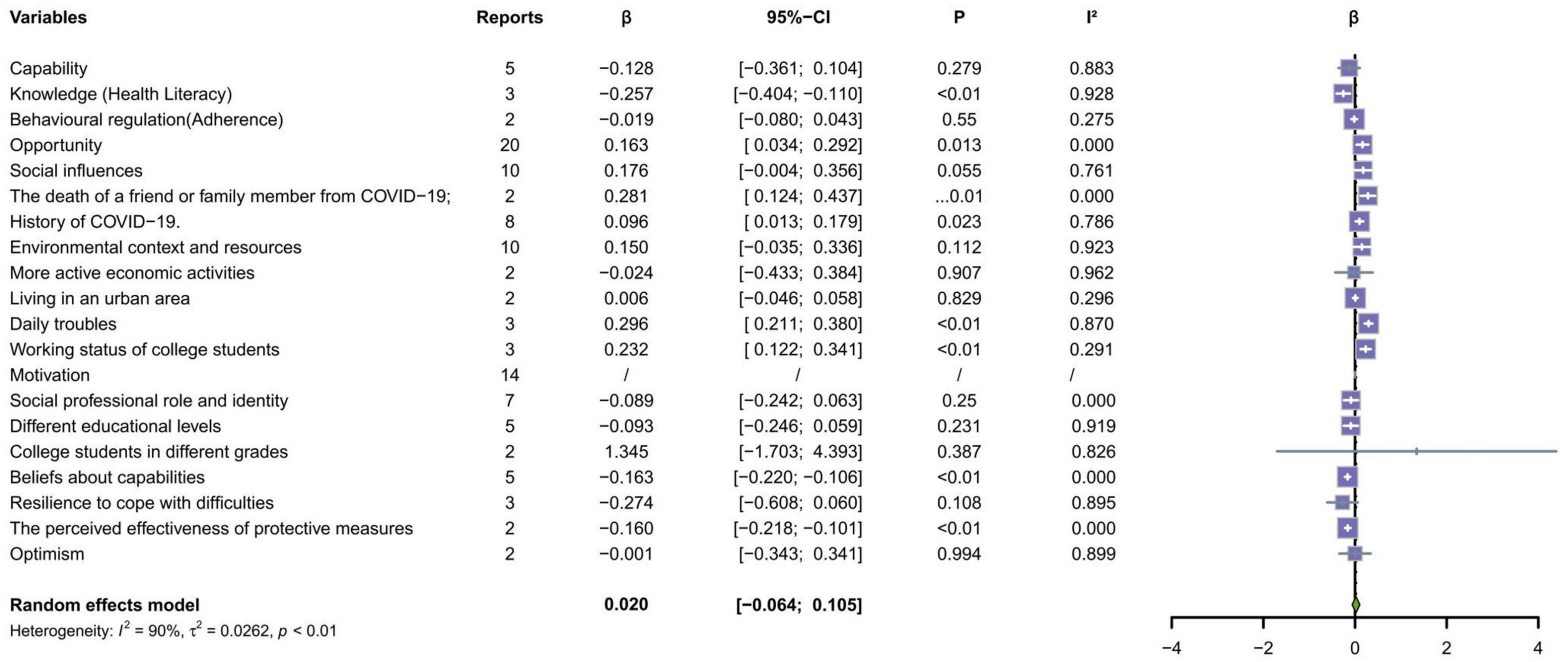
Meta-analysis of factors associated with pandemic fatigue (capability, opportunity, motivation) (*β*).

As shown in Figure 3, no significant association was found between capability and pandemic fatigue after merging the capability categories (*p* > 0.05). This study included only two TDF domains: knowledge and behavioral regulation. In the knowledge domain, there was a significant negative association between health literacy and pandemic fatigue (*β* = −0.257, 95%CI: −0.404, −0.110).

Figure 3 also showed a significant association between opportunity and pandemic fatigue (*β* = 0.163, 95%CI: 0.034, 0.292). In this study, the two TDF domains were social influences, environmental contexts and resources. In terms of social influences, the meta-analysis indicates that the association between social influences and pandemic fatigue is marginally significant (*p* = 0.055). It should be noted that individuals who had experienced the death of relatives and friends due to COVID-19 exhibited a marked increase in pandemic fatigue (*β* = 0.281,95%CI: 0.124, 0.437). Additionally, those with a history of COVID-19 showed a significant increase in pandemic fatigue (*β* = 0.096, 95%CI: 0.013, 0.179). Regarding the environmental context and resources, daily troubles (*β* = 0.296, 95%CI:0.211, 0.380) and the working status of college students (*β* = 0.232, 95%CI: 0.122–0.341) were significantly correlated with higher levels of pandemic fatigue.

When exploring motivation, this category cannot be combined due to the inability to convert *β* and OR values, and each domain needs to be discussed separately. This study identified five TDF domains that may be associated with pandemic fatigue: social-professional roles and identity, beliefs about capabilities, optimism, beliefs about consequences, and emotions.

**Figure 4:**
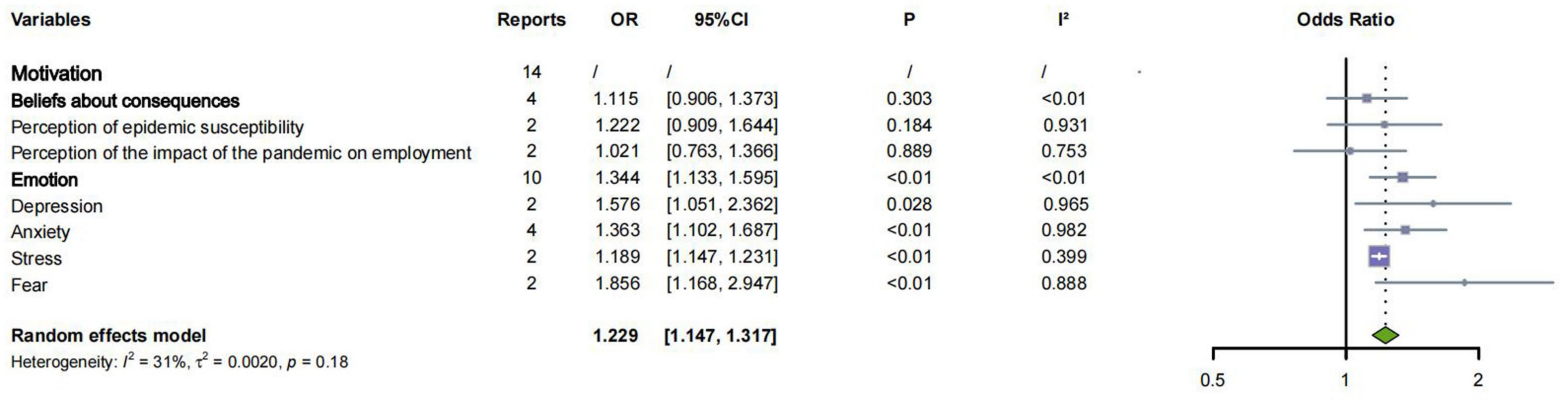
Meta-analysis of factors associated with pandemic fatigue (motivation) (OR).

Within these domains, as shown in Figure 3, we found that greater belief in the effectiveness of protective measures had significantly lower levels of pandemic fatigue (*β* = −0.160, 95%CI: −0.219, −0.102) in terms of beliefs about capabilities. Among the emotion-related studies, as shown in Figure 4, we explored depression (two studies), anxiety (four studies), stress (two studies), and fear (two studies). All these emotions were significantly associated with pandemic fatigue (*p* < 0.05). A meta-analysis found that individuals with negative emotions were more likely to experience pandemic fatigue (OR = 1.344, 95% CI: 1.133, 1.595). Pooled analyses of the other domains showed no significant association with pandemic fatigue.

Overall, findings point to an interdependent system in which cognitive capacities (health literacy), contextual stressors, and affective states jointly shape pandemic fatigue—an observation developed further in the Discussion.

## Discussion

Rather than viewing the COM-B components as independent factors, our findings suggest that pandemic fatigue emerges through recursive interactions among capability, opportunity, and motivation, forming a self-reinforcing behavioral system.

In this study, we found that the pandemic fatigue rate was not low. Based on the COM-B model, we systematically explored factors associated with pandemic fatigue from three perspectives: capability, opportunity, and motivation. In summary, the level of health literacy capability, experience of COVID-19 illness and death of individuals around them, daily troubles, and work status of college students cannot be ignored vis-à-vis pandemic fatigue. This meta-analysis refined the existing research system and aimed to identify and explore the current status of pandemic fatigue and its associated factors to provide a basis for further research.

According to the results of the meta-analysis, the current overall prevalence of broadly defined pandemic fatigue in adults worldwide is 51% (95%CI: 0.38, 0.65), and according to our findings, there is a substantial burden of pandemic-related fatigue manifestations among adults worldwide that is, there is widespread fatigue in the population in response to the pandemic. Most studies have also mentioned that this should be considered in subsequent public health policymaking (MacKay et al., 2022; Calleja et al., 2021; Leach et al., 2023). At the same time, our subgroup analysis also appeared to show that the prevalence was higher in the United States than in other countries, which may be related to the cultural context of the United States. However, this finding is exploratory and should not be overinterpreted. In societies with a culture of individualism, individual freedom and autonomy are highly valued, which can lead to increased public resistance to health guidelines and restrictions during a pandemic, resulting in higher feelings of fatigue (Demirtaş-Madran, 2021).

In terms of capability, further analysis revealed that within the domain of knowledge, health literacy—particularly regarding relevant information and measures during the pandemic—was closely related to pandemic fatigue; namely, high levels of health literacy could help individuals better understand and comply with preventive measures (Marquès-Pellejà et al., 2023; Mashinini et al., 2024), thereby reducing psychological stress and fatigue. At this point, higher health literacy was associated with lower pandemic fatigue: individuals with higher literacy were less prone to fatigue—likely because they can more effectively filter, appraise, and act on pandemic information. Therefore, public health education should focus on improving public health literacy, popularizing scientific health knowledge and protective measures, and enhancing public health awareness.

In terms of opportunities, the results suggest that pandemic fatigue may be higher when opportunities are affected. With regard to social influences, pandemic fatigue is now found to be marginally significant (*p* = 0.055). However, the overall relationship between opportunity and pandemic fatigue remains significant. With regard to social influences, pandemic fatigue is higher when individuals have experienced the death of a family member/close friend from COVID-19 or when they and their families/close friends have a history of COVID-19. This finding highlights the profound impact of bereavement on an individual’s mental health (Pearce et al., 2021). The loss of someone close not only takes a huge emotional toll but also increases an individual’s sense of fear and helplessness about the outbreak and increases the risk of pandemic fatigue (Kumar, 2023; Hu et al., 2023; Szczuka et al., 2023). Studies have also shown that witnessing or even experiencing the direct effects of COVID-19 can increase an individual’s psychological burden and anxiety, leading to increased pandemic fatigue (Abdul Rashid et al., 2023; Bae et al., 2024). Public health strategies should, therefore, include psychological support and counseling for those with a history of COVID-19 or those who have lost loved ones to help them survive and mitigate the impact of pandemic fatigue. In terms of the environmental context and resources, the working status of college students and daily troubles were closely related to pandemic fatigue. This finding is innovative because the relationship between these two factors and pandemic fatigue has rarely been discussed in the existing literature. Working students faced more challenges during the pandemic. Working students need to balance time and energy under the dual pressures of study and work (Li et al., 2022). Not only do they need to complete academic tasks, but they also need to take on work responsibility, which makes their daily lives busy (Yuebo et al., 2024; Christopher, 2018). This multitasking environment makes them more prone to fatigue and stress, especially during the pandemic (Marzouk et al., 2022). Moreover, they may lack emotional and social support systems available to full-time students during campus activities (Matz et al., 2023). In addition, the impact of socioeconomic changes during the pandemic on working students may have been more pronounced, such as reduced working hours or unemployment (Butter et al., 2022; Scelza et al., 2023; Li et al., 2015). Therefore, schools and governments should provide more financial subsidies and flexible arrangements for such students. Regarding daily troubles, changes in the pace of life, economic stress, and social isolation brought about by the pandemic have increased individuals’ daily troubles, thus exacerbating feelings of fatigue (Li et al., 2021). During the pandemic, many people faced family conflicts, challenges of working remotely, and rising costs of living. The long-term accumulation of these daily troubles tends to increase pandemic fatigue in individuals, thus affecting their compliance with epidemic prevention measures and psychological resilience to COVID-19-related information (Yan et al., 2022; Zhang et al., 2022; Chen et al., 2023; Moccia et al., 2020). Therefore, communities should strengthen their support networks to help residents cope with the annoyance in daily life. These findings highlight that pandemic fatigue is not merely an individual problem but a socially and structurally embedded outcome. Intervention strategies should therefore include structural supports—mental health services, social safety nets, and risk communication that acknowledges loss and grief—rather than only individual education.

Our findings suggest that negative motivation was associated with higher levels of fatigue during the pandemic. Depression, anxiety, stress, and fear were associated with higher levels of pandemic fatigue. Numerous studies have shown that negative emotions are prevalent during the pandemic and have become a common psychological burden in many people’s daily lives (AlKandari et al., 2022; Bai et al., 2024; Tang and He, 2023). Our study suggests that these key psychodynamic factors may contribute to the development of pandemic fatigue. However, it is important to note that there is some collinearity or conceptual overlap between negative emotions and motivation in the subjective experience of fatigue, which may have influenced the results. For example, anxiety may prompt individuals to engage in avoidance behaviors, which in turn aggravate anxiety (Deng et al., 2023; Gu et al., 2022). Depression can stem from loneliness due to social isolation, as well as anxiety about health and finances (Wu et al., 2017; Xu and Yan, 2022). During a pandemic, individuals’ subjective fear of possible infection will further exacerbate psychological stress, and stress with poor resilience will, in turn, produce fear (Husky et al., 2022; Singh et al., 2024; Chi et al., 2022; Xin et al., 2024). Depressed moods can lead to decreased energy, further leading to increased pessimism and increased fatigue with public health messages (Hoegh Poulsen et al., 2016; Jiang et al., 2023), while fear not only weakens an individual’s mental resilience but is also closely associated with excessive attention to and misunderstanding of information, which increases stress and leads to pandemic fatigue (Batmaz and Meral, 2022; Cai et al., 2020; Xin et al., 2022; Tan et al., 2020). Motivation functions both as an antecedent and consequence of fatigue. Negative emotions (depression, anxiety, stress, fear) amplify disengagement, while protective beliefs and resilience reduce fatigue. Notably, prolonged uncertainty promotes decision fatigue—continuous cognitive effort weakens self-regulation and willingness to act. Thus, interventions that bolster emotional resilience and reduce cognitive burden (clear messaging, simplified behavioral guidance) can help sustain motivation.

Integrating these findings, pandemic fatigue can be conceptualized as a behavioral depletion loop within the COM-B framework, where reduced capability may increases vulnerability to contextual stressors and may contribute to motivational erosion and eventual disengagement. Recognizing this reciprocity shifts the focus from single-factor interventions to multi-level approaches that simultaneously increase capability, expand opportunity, and replenish motivation.

Practically, this integrated interpretation suggests three complementary intervention targets: (1) Capability: deliver tailored health literacy training using age-appropriate formats; (2) Opportunity: strengthen community and institutional supports (bereavement counseling, accessible services, trusted communication channels); (3) Motivation: design communications that enhance perceived efficacy of measures, reduce cognitive load, and provide psychosocial resources to restore resilience. Such multi-component strategies align with the Behavior Change Wheel and are consistent with system-oriented approaches recommended in contemporary literacies.

### Strengths

This study consolidates and quantifies international evidence on pandemic fatigue and, for the first time to our knowledge, maps psychosocial factors systematically onto the COM-B/TDF framework. Strengths include a comprehensive search across English and Chinese databases, explicit coding of psychosocial factors, and synthesis of both prevalence and correlational evidence. By integrating cognitive, contextual, and affective factors, the analysis provides a theory-driven foundation for multi-level interventions.

### Limitations

Findings should be interpreted in light of several limitations. First, all included studies were cross-sectional, limiting causal inference and temporal interpretation of fatigue dynamics. As a result, while the analysis identifies factors associated with pandemic fatigue, causal inferences cannot be drawn, and these factors should not be interpreted as determinants but rather as potential correlates. Second, despite applying a random-effects model to account for variability across studies, substantial heterogeneity remained due to differences in measurement instruments, operational definitions of pandemic fatigue, and study methodologies. This variability may have constrained comparability and should be considered when interpreting the findings. Third, many studies relied on convenience sampling and online surveys, which may limit generalizability. Fourth, mixed reporting metrics (*β* vs. OR) prevented uniform pooling for some constructs, particularly motivational domains. In addition, *β*-based synthesis may have been constrained by differences in covariate adjustment and variable operationalization across studies, even though ORs and *β* coefficients were analyzed separately and unstandardized *β* coefficients were preferentially extracted. In particular, to ensure the consistency of the data, residual confounding cannot be ruled out. In addition, differences in variable operationalization and measurement scales may further limit direct comparability. In addition, the prevalence meta-analysis included only nine studies, and some subgroup estimates were based on one or two studies, so these findings should be regarded as exploratory. Similarly, publication bias testing had limited power under such conditions and should be interpreted cautiously. Finally, as the definition and scope of pandemic fatigue varied across studies, the inclusion criteria were based on a broad operational definition. In particular, some included studies emphasized information-related fatigue, whereas others focused more on behavioral disengagement or more generalized exhaustion, which may have contributed to conceptual heterogeneity across studies. Because the included prevalence studies used instruments with differing construct coverage and psychometric properties, direct comparability was inherently limited. Therefore, the pooled prevalence should be interpreted as an approximate estimate of the burden of broadly defined pandemic-related fatigue manifestations rather than the precise prevalence of a psychometrically uniform construct. Future longitudinal research with harmonized measures is needed to test the dynamic COM-B pathway, evaluate causal relationships, and assess targeted interventions.

## Conclusion

Pandemic fatigue is common and was associated with diverse capability-, opportunity-, and motivation-related factors. Interventions should address cognitive capacity (health literacy), mitigate contextual stressors (social and environmental supports), and sustain motivation (resilience and perceived efficacy). A COM-B-informed interpretation suggests multi-level strategies that combine skills building, supportive environments, and psychosocial support.
