# Supplementary material for: Pandemic fatigue and associated factors: a meta-analysis using the COM-B model
Source: Front Psychol. 2026 Apr 23;17:1765375. doi: 10.3389/fpsyg.2026.1765375 (PMC13149464; doi:10.3389/fpsyg.2026.1765375)
Supplement: Supplementary file 1 [file Supplementary_file_1.docx]

**Table S1.** Results of quality assessment of cross-sectional studies on the criteria of AHQR in the analysis

| **Study** | **1** | **2** | **3** | **4** | **5** | **6** | **7** | **8** | **9** | **10** | **11** | **score** |
| --- | --- | --- | --- | --- | --- | --- | --- | --- | --- | --- | --- | --- |
| Liu Yanni, 2023 | 1 | 0 | 1 | 1 | 0 | 0 | 0 | 0 | 0 | 1 | 0 | 4 |
| Mohd Radzniwan Abdul Rashid, 2023 | 1 | 0 | 1 | 0 | 1 | 0 | 0 | 0 | 0 | 1 | 0 | 4 |
| Ling Xin, 2022 | 1 | 1 | 1 | 0 | 1 | 0 | 0 | 0 | 0 | 1 | 1 | 6 |
| Yiqing Zhang, 2024 | 1 | 1 | 1 | 0 | 1 | 1 | 0 | 1 | 0 | 1 | 0 | 7 |
| Hiu Tin Leung, 2022 | 1 | 1 | 1 | 0 | 1 | 1 | 0 | 1 | 0 | 0 | 0 | 6 |
| Mengfei Guan, 2022 | 1 | 1 | 1 | 0 | 1 | 1 | 1 | 0 | 0 | 1 | 0 | 7 |
| Chenyuan Qin, 2023 | 1 | 1 | 1 | 0 | 1 | 1 | 1 | 1 | 0 | 1 | 0 | 8 |
| Xiaoquan Zhao, 2024 | 1 | 1 | 1 | 0 | 1 | 1 | 0 | 1 | 1 | 0 | 0 | 7 |
| Elsie Yan, 2022 | 1 | 1 | 1 | 0 | 1 | 1 | 1 | 0 | 0 | 1 | 0 | 7 |
| Jerome V. Cleofas, 2021 | 1 | 0 | 1 | 0 | 1 | 0 | 0 | 0 | 0 | 1 | 1 | 5 |
| Jerome V. Cleofas, 2021 | 1 | 0 | 1 | 1 | 1 | 1 | 0 | 1 | 0 | 1 | 0 | 7 |
| Jerome Cleofas, 2022 | 1 | 0 | 1 | 0 | 0 | 0 | 1 | 0 | 0 | 1 | 0 | 4 |
| Antonio González-Herrera, 2022 | 1 | 0 | 1 | 0 | 0 | 1 | 0 | 0 | 0 | 1 | 1 | 5 |
| Noha S Hassanien, 2022 | 1 | 0 | 1 | 1 | 1 | 0 | 0 | 0 | 0 | 1 | 0 | 5 |
| Rajesh Kumar, 2022 | 1 | 0 | 1 | 0 | 0 | 0 | 1 | 0 | 0 | 1 | 0 | 4 |
| Leodoro J Labrague, 2021 | 1 | 0 | 1 | 1 | 1 | 0 | 0 | 0 | 0 | 1 | 0 | 5 |
| Daniel W L Lai, 2023 | 1 | 0 | 1 | 0 | 1 | 0 | 0 | 0 | 0 | 1 | 0 | 4 |
| A. Haktanir，2022 | 1 | 0 | 1 | 0 | 1 | 1 | 1 | 1 | 0 | 0 | 0 | 6 |
| Jie Zhuang, 2025 | 1 | 0 | 1 | 1 | 1 | 1 | 0 | 1 | 0 | 0 | 0 | 6 |
| Nada Kotti, 2025 | 1 | 0 | 1 | 0 | 1 | 0 | 1 | 0 | 0 | 1 | 0 | 5 |
| Leiter, N , 2025 | 1 | 0 | 1 | 1 | 1 | 0 | 0 | 1 | 0 | 0 | 0 | 5 |
| Shuxin Yu, 2025 | 1 | 1 | 1 | 0 | 1 | 0 | 0 | 1 | 0 | 1 | 0 | 6 |
| Kexin Wang, 2026 | 1 | 1 | 1 | 0 | 1 | 1 | 0 | 0 | 1 | 1 | 0 | 7 |

TableS2. Detailed search strategies for all databases

The search strategy was developed from two main considerations: (1) pandemic fatigue and related fatigue manifestations, and (2) the pandemic context. To maximize sensitivity, broad fatigue-related terms were used at the database level. Synonyms within the fatigue-related concept were combined using the Boolean operator OR. Phrase searching with quotation marks was used for English-language databases, and database-specific field settings were adapted as appropriate (e.g., topic, title/abstract, or title/abstract/keywords). No truncation was used. Relevance to COVID-19 and related pandemics was further confirmed during title/abstract screening and full-text eligibility assessment.

General note

The literature search was conducted from January 1, 2020 to March 11, 2026. To maximize sensitivity, broad fatigue-related terms were used at the search stage. Relevance to COVID-19 and related pandemics was further determined during title/abstract screening and full-text eligibility assessment.

1. China National Knowledge Infrastructure (CNKI)

Search field: 主题

Search strategy:

（“疫情疲劳” OR“大流行疲劳” OR “疫苗疲劳” OR “信息疲劳” OR“行为疲劳” OR “免疫疲劳”）

Time limit: January 1, 2020 to March 11, 2026

2. Wanfang Database

Search field: 主题

Search strategy:

（“疫情疲劳” OR“大流行疲劳” OR “疫苗疲劳” OR “信息疲劳” OR “行为疲劳” OR “免疫疲劳”）

Time limit: January 1, 2020 to March 11, 2026

3. PubMed

Search field: Title/Abstract

Search strategy:

("Pandemic Fatigue"[Title/Abstract] OR "Vaccine Fatigue"[Title/Abstract] OR "Information Fatigue"[Title/Abstract] OR "Behavioral Fatigue"[Title/Abstract] OR "Immune Fatigue"[Title/Abstract] OR "Message Fatigue"[Title/Abstract])

Time limit: January 1, 2020 to March 11, 2026

4. Embase

Search field: Title/Abstract/Keyword

Search strategy:

('pandemic fatigue':ti,ab,kw OR 'vaccine fatigue':ti,ab,kw OR 'information fatigue':ti,ab,kw OR 'behavioral fatigue':ti,ab,kw OR 'immune fatigue':ti,ab,kw OR 'message fatigue':ti,ab,kw)

Time limit: January 1, 2020 to March 11, 2026

5. Web of Science

Search field: Topic

Search strategy:

TS=("Pandemic Fatigue" OR "Vaccine Fatigue" OR "Information Fatigue" OR "Behavioral Fatigue" OR "Immune Fatigue" OR "Message Fatigue")

Time limit: January 1, 2020 to March 11, 2026

6. Scopus

Search field: Title, Abstract, Keywords

Search strategy:

TITLE-ABS-KEY("Pandemic Fatigue" OR "Vaccine Fatigue" OR "Information Fatigue" OR "Behavioral Fatigue" OR "Immune Fatigue" OR "Message Fatigue")

Time limit: January 1, 2020 to March 11, 2026

Notes on screening

Studies retrieved through the above search strategies were further screened according to the predefined eligibility criteria. Although the search strategy used broad fatigue-related terms to ensure sensitivity, only studies relevant to COVID-19 and related pandemics and meeting the operational definition of pandemic fatigue were included in the final review.

TableS3. Influencing factors of pandemic fatigue (based on COM-B framework and TDF theory)

| **COM-B component** |  | **TDF domain with definition** | **Influencing factors** |
| --- | --- | --- | --- |
| **Capability**  the individual’s psychological and  physical capacity to engage in the  activity concerned | **1.Psychological**  Knowledge or psychological skills, strength or stamina to engage in the necessary mental processes;  **2.Physical**  Physical skill, strength or stamina. | 1.Knowledge  Awareness of the existence of something | Health literacy of individuals accessing, understanding and applying health information |
|  |  | **2.Skills**  Ability or proficiency acquired through practice | Not mentioned in the included studies. |
|  |  | **3.Memory, attention and decision processes**  The ability to retain information, focus selectively on aspects of the environment and choose between two or more alternatives | Not mentioned in the included studies. |
|  |  | 4.Behavioural regulation  Anything aimed at managing or changing objectively observed or measured actions | Adherence to control measures during a pandemic. |
| **Opportunity**  all the factors that lie outside the individual that make the behavior possible or prompt it | **1.Social Opportunity**  afforded by interpersonal influences, social cues and cultural norms that influence the way that we think about things;  **2.Physical Opportunity**  afforded by the environment  involving time, resources, locations, cues, physical “affordance”. | **5.Social influences**  Interpersonal processes that can cause an individual to change their thoughts, feeling or behaviors | The death of a friend or family member from COVID-19;  History of COVID-19. |
|  |  | 6.Environmental context and resources  Any circumstance of a person’s situation or environment that discourages or encourages the development of skills and abilities, independence, social competence, and adaptive behavior | The level of economic activity;  Living in an urban area (resource differences affect how individuals perform protective behaviors);  All kinds of small troubles in daily life;  Working status of college students (working conditions and environment during the epidemic will affect compliance with protective measures). |
| **Motivation**  all those brain processes that energize and direct behavior, not just goals and conscious decision-making | **1.Reflective**  Involving plans (self-conscious intentions) and evaluations (beliefs about what is good and bad);  **2.Automatic**  Involving emotional reactions, desires (wants and needs), impulses, inhibitions, drive states and reflex responses. | 7.Social professional role and identity  A coherent set of behaviors and displayed personal qualities of an individual in a social or work setting | Individuals with different educational levels assume different roles in the society;  College students have different social roles in different grades. |
|  |  | **8.Beliefs about capabilities**  Acceptance of the truth/reality about or validity of an ability, talent or facility that a person can put to constructive use. | Resilience that reflects the individual's confidence and ability to cope with difficulties;  The perceived effectiveness of protective measures during a pandemic. |
|  |  | 9.Optimism  Confidence that things will happen for the best or that desired goals will be attained | Optimism about the pandemic. |
|  |  | 10.Beliefs about consequences  Acceptance of the truth/reality about or validity of outcomes of a behavior in a given situation | Perception of epidemic susceptibility;  Perception of the impact of the pandemic on employment. |
|  |  | 11.Reinforcement  Increasing the probability of a response by arranging a dependent relationship, or contingency, between the response and a given stimulus | Not mentioned in the included studies. |
|  |  | **12.Intentions**  Conscious decision to perform a behavior or a resolve to act in a certain way | Not mentioned in the included studies. |
|  |  | 13.Goals  Mental representation of outcomes or end states that an individual wants to achieve | Not mentioned in the included studies |
|  |  | 14.Emotion  A complex reaction pattern, involving experiential, behavioral and physiological elements, by which the individual attempts to deal with a personally significant matter or event | Depression about control measures and information during the pandemic;  Anxiety about containment measures and information during the pandemic;  Feeling stressed when exposed to containment measures and information during the pandemic;  Fear of COVID-19 and its information and control measures. |

TableS4. Meta-analysis of influencing factors and pandemic fatigue (Capacity)

| **Variables** | **Reports** | **β (95% CI)** | **P(association)** | **I**2**(%)（heterogeneity）** | **P（heterogeneity）** | **P (begg’s test)** |
| --- | --- | --- | --- | --- | --- | --- |
| **Capability** | **5** | **-0.128（-0.361~**  **0.104）** | **0.279** | **88.3** | **0.063** | **1.000** |
| **Knowledge** | **3** | **-0.257（-0.404~**  **-0.110）** | **<0.01** | **92.8** | **<0.000** | **1.000** |
| Health literacy of individuals accessing, understanding and applying health information |  |  |  |  |  |  |
| **Behavioral regulation** | **2** | **-0.019 (-0.080**  **~0.043)** | **0.550** | **27.5** | **0.240** | **1.000** |
| Adherence |  |  |  |  |  |  |

TableS5. Meta-analysis of influencing factors and pandemic fatigue (Opportunity)

| **Variables** | **Reports** | **β (95% CI)** | **P(association)** | **I**2**(%)（heterogeneity）** | **P（heterogeneity）** | **P (begg’s test)** |
| --- | --- | --- | --- | --- | --- | --- |
| **Opportunity** | **20** | **0.163（0.034~**  **0.292）** | **0.013** | **0.0** | **<0.000** | **0.707** |
| **Social influences** | **10** | **0.176(-0.004~**  **0.356)** | **0.055** | **76.1** | **0.089** | **1.000** |
| The death of a friend or family member from COVID-19 | 2 | 0.281(0.124~  0.437) | <0.01 | 0.0 | 0.710 | 1.000 |
| History of COVID-19 | 8 | 0.096(0.013~  0.179) | 0.023 | 78.6 | <0.001 | 1.000 |
| **Environmental context and resources** | **10** | **0.150(-0.035~**  **0.336)** | **0.112** | **92.3** | **<0.000** | **1.000** |
| The level of economic activity | 2 | -0.024(-0.433~  0.384) | 0.907 | 96.2 | <0.001 | 1.000 |
| Living in an urban area | 2 | 0.006(-0.046  ~0.058) | 0.829 | 29.6 | 0.233 | 1.000 |
| Daily troubles | 3 | 0.296(0.211~  0.380) | <0.01 | 87.0 | 0.005 | 1.000 |
| Working status of college students | 3 | 0.232(0.122~  0.341) | <0.01 | 29.1 | 0.244 | 1.000 |

TableS6. Meta-analysis of influencing factors and pandemic fatigue (Motivation)(β)

| **Variables** | **Reports** | **β (95% CI)** | **P(association)** | **I**2**(%)（heterogeneity）** | **P（heterogeneity）** | **P (begg’s test)** |
| --- | --- | --- | --- | --- | --- | --- |
| **Motivation** | **14** | **/** | **/** | **/** | **/** | **/** |
| **Social Professional role and identity** | **7** | **-0.089(-0.242~**  **0.063)** | **0.250** | **0.0** | **0.356** | **1.000** |
| Different educational levels | 5 | -0.093(-0.246~  0.059) | 0.231 | 91.9 | <0.001 | 0.806 |
| College students in different grades | 2 | 1.345(-1.703~  4.393) | 0.387 | 82.6 | 0.017 | 1.000 |
| **Beliefs about capabilities** | **5** | **-0.163(-0.220~**  **-0.106)** | **<0.01** | **0.0** | **0.337** | **1.000** |
| Resilience to cope with difficulties | 3 | -0.274(-0.608~  0.060) | 0.108 | 89.5 | <0.001 | 1.000 |
| The perceived effectiveness of protective measures | 2 | -0.160(-0.218~  -0.101) | <0.01 | 0.0 | 0.828 | 1.000 |
| **Optimism** | **2** | **-0.001(-0.343~**  **0.341)** | **0.994** | **89.9** | **0.002** | **1.000** |

TableS7. Meta-analysis of influencing factors and pandemic fatigue (Motivation)(OR)

| **Variables** | **Reports** | **OR (95% CI)** | **P(association)** | **I**2**(%)（heterogeneity）** | **P（heterogeneity）** | **P (begg’s test)** |
| --- | --- | --- | --- | --- | --- | --- |
| **Motivation** | **14** | **/** | **/** | **/** | **/** | **/** |
| **Beliefs about consequences** | **4** | **1.115(0.906~**  **1.373)** | **0.303** | **0.0** | **0.397** | **1.000** |
| Perception of epidemic susceptibility | 2 | 1.222(0.909~  1.644) | 0.184 | 93.1 | <0.000 | 1.000 |
| Perception of the impact of the pandemic on employment | 2 | 1.021(0.763~  1.366) | 0.889 | 75.3 | 0.044 | 1.000 |
| **Emotion** | **10** | **1.344(1.133~**  **1.595)** | **<0.01** | **55.8%** | **0.079** | **0.089** |
| Depression | 2 | 1.576(1.051~  2.362) | 0.028 | 96.5 | <0.000 | 1.000 |
| Anxiety | 4 | 1.363(1.102~  1.687) | 0.004 | 98.2 | <0.000 | 1.000 |
| Stress | 2 | 1.189(1.147~  1.231) | <0.01 | 39.9 | 0.197 | 1.000 |
| Fear | 2 | 1.856(1.168~  2.947) | 0.009 | 88.8 | 0.003 | 1.000 |
